# Imaging and genetic toolbox to study *Arabidopsis* embryogenesis

**DOI:** 10.1111/nph.71072

**Published:** 2026-03-11

**Authors:** David Babić, Milan Župunski, Jiří Friml

**Affiliations:** ^1^ Institute of Science and Technology Austria (ISTA) 3400 Klosterneuburg Austria; ^2^ Institute of Cell and Interaction Biology Heinrich Heine Universität 40225 Düsseldorf Germany

**Keywords:** *Arabidopsis thaliana*, auxin, cell division orientation, embryogenesis, live‐cell imaging, zygote polarity

## Abstract

Embryogenesis in the model plant *Arabidopsis thaliana* provides a framework for understanding how cell polarity and patterning coordinate with hormonal signalling to establish the plant body plan. Following fertilisation, the zygote divides asymmetrically to generate apical and basal lineages, establishing the apical–basal axis that defines future shoot and root poles. Genetic and molecular analyses of classical mutants including *gnom*, *monopteros (mp)*, *bodenlos (bdl)* and *topless* revealed that localised auxin biosynthesis, directional transport and downstream transcriptional responses are central to apical–basal axis establishment and organ initiation. The main components of this regulation are polarly localised PIN auxin transporters and downstream modules involving MONOPTEROS and WUSCHEL‐RELATED HOMEOBOX transcription factors. Advances in microscopy have transformed the study of *Arabidopsis* embryogenesis: fluorescence‐compatible clearing reagents and three‐dimensional reconstructions now permit quantitative analyses of cell geometry, division orientation, and cytoskeletal dynamics. Live ovule imaging setups with confocal laser scanning and multiphoton microscopes enable real‐time observation of embryo development, while laser‐assisted cell ablation can be used to probe cell‐to‐cell communication and fate plasticity. Together, these methodological breakthroughs position *Arabidopsis* embryos as a prime model for dissecting the chemical and biophysical cues that shape plant development.

## Progress of *Arabidopsis* embryogenesis

**Fig. 1:**
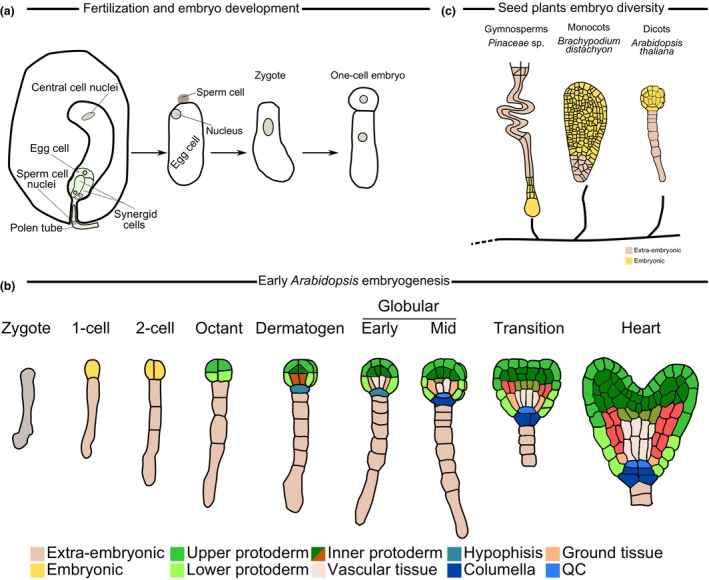
Aspects of plant embryogenesis – fertilisation, evolutionary comparisons, and early *Arabidopsis thaliana* embryo patterning. (a) The plant gametophytes (female in the ovule; male in the pollen tube), general process of fertilisation, formation of the zygote, and the emergence of the early embryo, based on these processes in *A. thaliana*. Inspired by and adapted from Maruyama *et al*. (2015). (b) Schematic representation of the variation between the body plan of different plant embryos. Colours indicate embryonic or extraembryonic tissue. Gymnosperm embryo adapted from Wardlaw (1955); monocot embryo adapted from Hao *et al*. (2021, under the CC‐BY 4.0 license). (c) The process of early *Arabidopsis* embryogenesis. The pattern progression is shown from the division of the zygote until the early heart stage, showing the most important events in the formation of the embryo body axes and pattern, including the emergence of different cell populations. The colours indicate the populations of cells according to their lineage. Inspired by and modified from Ten Hove *et al*. (2015, under the CC‐BY 4.0 license).

Plants, like animals, begin their life as a single cell – the zygote. The zygote is the result of fertilisation of the egg cell by the sperm cell. The zygote undergoes a series of cell divisions, which establish the plant embryo (Fig. 1a). Although plant embryo development and patterning vary greatly throughout the plant kingdom (Radoeva *et al*., 2019) (Fig. 1b), embryogenesis of the model species *Arabidopsis thaliana* L. (Brassicaceae) follows a trajectory of highly predictable cell divisions and differentiations (Fig. 1c). The stereotypic pattern of cell divisions and expansions and power of *Arabidopsis* genetics have led the majority of plant embryo research to focus on this model (Meinke, 2020; Harnvanichvech *et al*., 2021; Gillmor, 2026).

*Arabidopsis* embryogenesis starts with an asymmetric transverse division of the zygote producing the basal and the apical daughter cells. The basal cell will form the extraembryonic suspensor, a support structure connecting the embryo with the maternal tissue. The apical cell will give rise to most cells of the proembryo and in turn the seedling (Ten Hove *et al*., 2015). This division of the zygote is the first key patterning event, establishing the apical–basal axis of the future plant body. The apical cell then undergoes two periclinal divisions to produce the 4‐cell embryo, and a second set of periclinal divisions to produce the 8‐cell proembryo with a proto‐epidermal layer and a newly radial body symmetry (De Smet *et al*., 2010; Wabnik *et al*., 2013). There is now a distinction between the protoderm and the ground tissue, and the radial pattern emerges, which later results in a series of concentric cylinders of different cell types of the postembryonic root (Fig. 1c). After another round of divisions, at the embryonic 32‐cell stage, the uppermost cell of the suspensor (the hypophysis) divides asymmetrically, giving rise to two daughter cells – an upper, lens‐shaped cell and a lower cell. These cells undergo an identity switch from an extra‐embryonic to embryonic and give rise to the future to the quiescent centre and the columella of primary root cap. Shortly afterwards, the upper edges of the globular embryo expand, resulting in the triangle‐shaped transition stage embryo with the emerging bilateral symmetry. Cotyledons are formed from continued outgrowth of the upper vertices of the triangle, giving rise to the aptly named heart stage embryo (Fig. 1c). The embryo elongates further to produce the torpedo and bent cotyledon stages where the embryo gradually acquires the shape of the future seedling.

Within this review, we aim to focus on imaging techniques that have shaped this research field. However, it is necessary to provide a brief overview of genetic studies, without which the development and application of imaging techniques would not be meaningful.

## Auxin‐centred genetic and hormonal mechanisms underlying plant embryogenesis

The initial work on *Arabidopsis* embryogenesis was inspired by studies performed in *Drosophila*; therefore, the early researchers followed a similar approach and performed genetic screens aimed at identifying mutants with embryo patterning defects (Jürgens *et al*., 1991; Barton & Poethig, 1993; Gillmor, 2026). The majority of our molecular mechanistic understanding of embryo patterning stems from this early genetic groundwork and the following molecular characterisation of the corresponding genes (reviewed in detail in Meinke, 2020). Notably, some of the most prominent mutants discovered by this approach, including *topless*, *monopteros*, *bodenlos*, and *gnom*, were later linked to signalling or transport of the phytohormone auxin (Berleth & Jürgens, 1993; Mayer *et al*., 1993; Hamann *et al*., 1999; Long *et al*., 2002; Möller & Weijers, 2009).

**Fig. 2:**
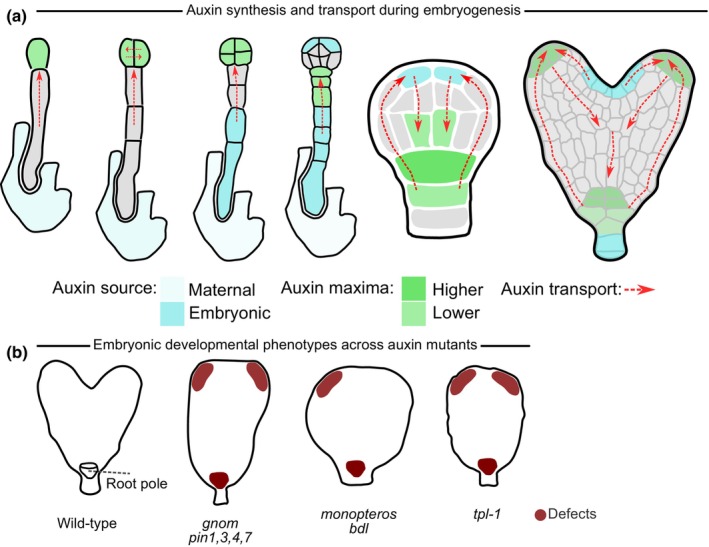
Role of auxin in *Arabidopsis thaliana* embryo development. (a) Directional auxin fluxes in early embryogenesis. Highlighted are the auxin sources (places of auxin biosynthesis – initial basal/maternal and later apical/embryonic) in shades of blue, auxin maxima (initial apical, later basal) in shades of green, and directions of auxin transport in red dashed arrows. (b) Schematic representations of some typical embryo patterning phenotypes in various mutants related to auxin transport, metabolism, or signalling. The burgundy‐coloured regions in the mutant embryo outlines refer to apical (cotyledon) and basal (root pole) patterning defects.

The role of auxin in plant embryogenesis has been studied in great detail in the last three decades, leading to a robust model of how this hormone contributes to the patterning of the plant embryo. Auxin is involved at the earliest stages of embryogenesis; likely synthesised in maternal tissues such as the ovule integuments and transported towards the base of the embryo (Robert *et al*., 2013, 2018). Auxin is then transported via the activity of the PIN7 auxin transporters in suspensor towards the early embryo apex where auxin accumulates and contributes to the specification of the apical cell identities (Friml *et al*., 2003; Robert *et al*., 2013). At around the 16‐cell embryo stage, a new auxin source emerges at the apex of the proembryo (Robert *et al*., 2013; Wabnik *et al*., 2013). The auxin synthesised in the uppermost protodermal cells is then directed via basally localised PIN1 through the proembryo interior towards its base, the so‐called root pole, where it accumulates, initiates transcriptional responses, and induces the formation of the root apical meristem (Schlereth *et al*., 2010) (Fig. 2a). Also, AUX1‐/LAX‐mediated auxin influx decisively contribute to embryo patterning (Robert *et al*., 2015) but its role is less clear.

These aspects of *Arabidopsis* embryo patterning and body organisation mentioned above are partially conserved across the plant kingdom (Radoeva *et al*., 2019). In lineages of land plants (Embryophyta), there exists a common delineation of embryonic/apical and extra‐embryonic/basal cell lineages, leading to apical–basal axis specification (Fig. 1b). Dynamics of local auxin flows such as emergence and maintenance of local auxin maxima via auxin transport are developmental modules, present throughout the plant kingdom and have been extensively studied in monocots, such as maize (Chen *et al*., 2014; Zhao *et al*., 2017; Robert *et al*., 2018; Dresselhaus & Jürgens, 2025) and *Brachypodium* (Hao *et al*., 2021). We respectfully acknowledge the importance of this work, but due to space constraints, we will focus on *Arabidopsis* embryo research.

The essential role of auxin, biosynthesis, transport, and transcriptional responses in embryo patterning has been shown by analysing embryonic phenotypes of mutants defective in these processes. Embryo patterning defects resulting from disrupting auxin mechanisms typically include defects in (i) early establishment of apical/basal cell boundary, for example, in *pin7* (Friml *et al*., 2003), *bdl* and *mp* (Hamann *et al*., 1999), or auxin biosynthesis mutants (Robert *et al*., 2018); (ii) root pole specification, for example, in multiple *pin* mutants (Friml *et al*., 2003) or in the PINOID overexpressor (Friml *et al*., 2004), *mp* or *bdl* (Hardtke & Berleth, 1998; Hamann *et al*., 2002); and (iii) cotyledon specification (in most of the mentioned mutants). Phenotypes of these well‐known mutants captured the attention of researchers, ultimately leading them to describe genetic and molecular processes underlying early embryo patterning (Fig. 2b). These early studies lay the groundwork for *Arabidopsis* embryo research and contributed to the advancement of the entire field of plant developmental biology, helping to establish auxin and its local distribution as a central, plant‐specific mechanism of patterning (Vanneste *et al*., 2025).

The somehow auxin‐centric bias of the *Arabidopsis* embryogenesis studies in late 1990s and 2000s reflected also in this chapter originates from unbiased findings that many early‐identified embryo and seedling patterning mutants (*topless*, *monopteros*, *bodenlos*, *gnom*) and embryo markers (*LENNY*/*PIN4*, *PIN7*) were all directly linked to transport and action of auxin. Nonetheless, there are also many other auxin‐independent key regulators involved in early embryogenesis. For example, polarity of the zygote and the establishment of the apical to basal polarity of the 1‐cell stage embryo require activity of the *WUSCHEL‐RELATED HOMEOBOX* (*WOX*) genes (Haecker *et al*., 2004; Breuninger *et al*., 2008; Ueda *et al*., 2011). Cell fate regulation of apical and basal cell and their subsequent tissue identities is mediated by WOX2 and WOX8, respectively, which are co‐expressed in the egg cell and zygote. After the first division of the zygote, WOX2 remains confined to the apical lineage and WOX8 to the (basal) suspensor cells (Haecker *et al*., 2004), manifesting a strong delineation between these two main developmental trajectories. Nonetheless, WOXs have also been linked to auxin‐mediated processes as they converge on the MP signalling pathway and affect PIN1 expression (Breuninger *et al*., 2008).

Other noteworthy mentions among dozens and dozens of embryo patterning mutants that contributed to our understanding of plant development include SHORT ROOT (SHR) and SCARECROW (SCR) regulators of radial and other patterning processes (Shaar‐Moshe *et al*., 2022), YODA, a constituent of a MITOGEN‐ACTIVATING PROTEIN (MAP) kinase signalling pathway involved in polarisation of the zygote (Lukowitz *et al*., 2004; Wang *et al*., 2021), and the PLETHORA transcription factors with their role in defining root identity (Smith & Long, 2010; Pires *et al*., 2024). In particular, the YODA–MPK3/6 signalling module‐mediated phosphorylation of the ICE1/SCRM transcription factor emerges as a mechanism acting in parallel to auxin and also converging on WOX transcription factors to regulate zygote polarisation and early apical–basal patterning (Chen *et al*., 2025).

All these insights have been revealed predominantly by genetic approaches combined with microscopic methods to characterise *Arabidopsis* embryogenesis and we have witnessed an amazing advancement of techniques and approaches to visually dissect these processes. Below, we discuss the most important methodological breakthroughs in visualising *Arabidopsis* embryo development.

## Clearing of plant embryos for microscopic analysis

**Fig. 3:**
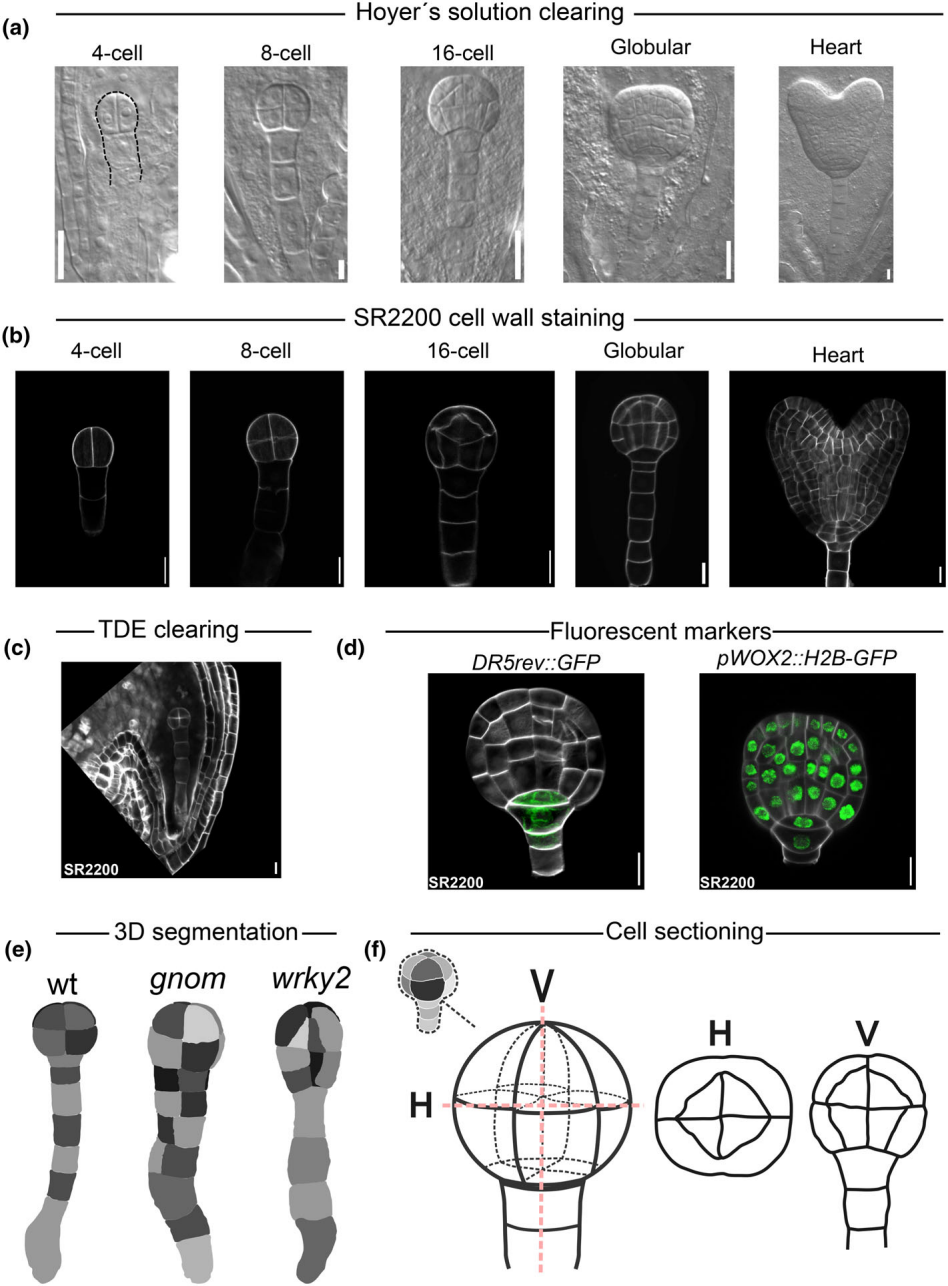
Microscopical and computational visualisation of *Arabidopsis thaliana* embryos. (a) Cleared embryos of indicated stages. Black dashed line outlines the 4‐cell stage embryo. (b) SCRI Rennaisance 2200 (SR2200) dye allows for embryonic cell outlines to be visible due to cell wall staining. Stages are indicated. (c) Combining SR2200 staining with 2,2′‐thiodiethanol (TDE) clearing allows for embryos to be inspected within the ovules. Cell walls in both are stained. (d) Different fluorescent tags can be visualised in concurrence with the cell wall staining. Left panel – ER‐localised DR5. Right panel – Nuclear‐localised WOX2. (e) Cell segmentation of whole embryos (stained with mPS‐PI) of indicated genotypes. Adapted and modified from Yoshida *et al*. (2014). (f) Examples of transverse (middle panel) and longitudinal (right panel) cross sections employed in 3D morphometry. H, horizontal; V, vertical. Bars, (a–d) 10 μm.

*Arabidopsis* embryos develop inside ovules inside siliques. Being embedded deep underneath several layers of maternal tissue complicates approaches to observe and analyse their development. In the early days, embryologists employed tissue sectioning methods (Mansfield & Briarty, 1991; Mayer *et al*., 1993) to document embryo shape, cell organisation and patterning. Later, a whole‐mount tissue clearing method, using a Hoyer's chloral‐hydrate‐based solution was applied (Berleth & Jürgens, 1993; Vernon & Meinke, 1994), allowing for easier observation of the embryo at any stage inside an intact ovule (Fig. 3a). This method became a benchmark in many ground‐breaking studies. As confocal microscopy became the standard imaging approach, efforts to optimise the clearing methods focused on preservation of fluorescence of reporter proteins and overcoming the limitations of light scattering. Over time, different clearing solutions for fluorescent imaging with varying utility have emerged. Chemicals such as ClearSee (Kurihara *et al*., 2015) or 2,2′‐thiodiethanol (TDE) (Musielak *et al*., 2016) have been employed to clear the ovular tissue layers to observe the embryos underneath. Recently, Attuluri *et al*. (2022) have compared different clearing methods, including modifications of original protocols, and scored them for different properties, such as clearing efficiency, changes in sample morphology, and spectral properties.

To study embryo patterning in detail, one needs to discern the outlines of individual embryonic cells, as cell wall orientation: a crucial information that indicates the cell division planes and shapes (De Smet & Beeckman, 2011). Researchers used dyes such as FM4‐64 to stain the plasma membrane or cell wall (Jelínková *et al*., 2010; Rademacher *et al*., 2011). However, these chemicals emit in the red part of the spectrum, which might interfere with other dyes and often cannot penetrate deep into tissue (Musielak *et al*., 2016). In the last decade, SCRI Rennaisance 2200 (SR2200) stain has often been used to stain the cell walls of the embryo (Fig. 3b). SR2200 is an optical brightener that binds to β‐glucans in the cell wall and has been used in fungi (Harris *et al*., 2002) and adapted for *Arabidopsis* embryos (Smith & Long, 2010; Robert *et al*., 2013; Wendrich *et al*., 2015). The protocol has been further modified to combine SR2200 with TDE clearing (Slane *et al*., 2017) allowing observation of embryos within intact ovules (Fig. 3c). SR2200 has an emission spectrum quite similar to DAPI, although the two can be spectrally unmixed (Musielak *et al*., 2015) and therefore can be combined with fluorescent proteins across the spectrum in confocal imaging. Musielak *et al*. (2015) have made a highly efficient and easy‐to‐use protocol which can be used on extracted embryos to visualise not only embryo patterning but also fluorescent reporter proteins in different subcellular compartments (Fig. 3d). As a testament to the efficacy of this protocol, Liao & Weijers (2018) have made a valuable collection of embryo‐specific markers that were imaged in different SR2200‐based mounting media and have shown the preservation of delicate subcellular structures when using this imaging approach.

## 3D morphometric analyses of *Arabidopsis* embryos

Use of SR2200 dye to stain *Arabidopsis* embryo cell walls allowed the study of embryo patterning in three dimensions (3D), changing our perspective from the typical, cleared 2D image, which cannot immediately discern between 2‐ and 4‐cell stage embryos using classical clearing methods (Fig. 3a). Staining with SR2200 allows measurement of cell volumes, as well as allowing insight into the orientations of the cell division planes (Fig. 3e,f). The segmentation of individual cells based on Z‐stacks of SR2200‐stained embryos from 1‐ to early globular stage embryos allowed generating a developmental map of embryogenesis (Yoshida *et al*., 2014). This approach helped to dissect cell division orientations during *Arabidopsis* embryogenesis, showing that patterns arise by avoiding the ‘shortest wall through the cell’ route, and that this deviation is triggered by auxin (Yoshida *et al*., 2014). A follow‐up work showed further that the orientation of the cell division can be predicted and seems to rely on the distance from the centroid of the mother cell, as well as the positioning of the nucleus (Moukhtar *et al*., 2019). This methodical approach has also been utilised to study the auxin response‐dependent organisation of cytoskeleton, which leads to asymmetric divisions and ultimately, embryonic cell geometry (Vaddepalli *et al*., 2021). Intriguingly, Chakrabortty *et al*. (2018) have shown that organisation of microtubule array orientation can predict division planes in embryos. Combining this method with large‐scale computational analysis, Laruelle *et al*. (2022) have provided an in‐depth inquiry into the variability in cell division patterns during embryogenesis, also hinting that maternal cell geometry could influence the patterning of the cell divisions. For more details on the growing field of embryo computational cell biology, we recommend Harnvanichvech *et al*. (2021). Advances like this indeed have been giving us completely novel insights into the most basic rules behind the patterning not only of *Arabidopsis* embryos but plant tissues in general, proving a value of this model system.

## Ovule cultures and pharmacological treatments

The methods we have discussed until this point are based on visualising fixed embryos either in intact ovules or extracted from them; none of these allow live imaging of embryos. Live imaging allows addressing processes elusive to the fixed samples, such as length of cell cycle, protein turnover, or transient cell responses to hormones and other cues. To achieve live imaging, one must establish a functional embryo or ovule culture as it seems not feasible to visualise embryos within intact siliques. Ovule culturing allows for the development of zygotic embryos in a biological context similar to that within an *Arabidopsis* silique. Somatic embryo culturing has been used in many studies, but somatic embryogenesis does not necessarily exactly recapitulate zygotic embryogenesis and depends on the medium composition as well as the origin of the tissue (Horstman *et al*., 2017; Antoshina *et al*., 2024). Sauer & Friml (2004) first described an *Arabidopsis* ovule culture with the sole focus on embryogenesis studies. The authors used a modified Murashige and Skoog medium, adding phytagel and glutamine, which allowed for normal embryogenesis from the 1‐cell stage until the adult plant. Afterwards, the ovule culturing medium was improved (Gooh *et al*., 2015), whereby the authors used a Nitsch medium‐based solution supplemented with trehalose, which likewise leads to full reconstruction of adult plants from *in vitro* grown ovules harbouring 1‐cell stage embryos. Of note, the ovules cultured in this medium showed higher survival rates, with most embryos following a normal development pattern. Although these approaches are still limited, the development of such relatively convenient ovule culturing methods has allowed the researchers to chemically influence cellular and developmental processes in order to dissect their contributions to embryogenesis. Treatments involved manipulating auxin transport, vesicular trafficking, and the cytoskeleton (Sauer & Friml, 2004; Kimata *et al*., 2016, 2019, 2020), or more recently, cell division (Kimata *et al*., 2023), highlighting the applicability of the approach. The development of such ‘pharmaceutical’ approaches is arguably a powerful tool to study mechanisms of embryogenesis and would undoubtedly open new venues and perspectives in plant embryo research.

## Live‐cell imaging of *Arabidopsis* embryos

The successful development of ovule culturing approaches as well as the remarkable advancement of microscopical tools has led to development of live‐cell imaging methods. Live‐cell imaging of various plant tissues offers direct observation of (sub)cellular dynamics at precise spatiotemporal scales and has provided unprecedented insights into different cellular and developmental processes. Moreover, it allows to follow more rigorously changes in cells and tissues following experimental manipulation. Unfortunately, plant embryos are a type of sample on which live‐cell imaging is rather difficult. Long *et al*. (2020) developed a methodological approach to perform protein–protein interactions in living heart‐stage *Arabidopsis* embryos. Despite this technique having potential to monitor protein interactions, it does not provide for observations of very early embryos nor for dedicated imaging over longer periods of time. Other notable example of live imaging, however, only on somatic embryos, yielded key insights into the mechanism of somatic cells reprogramming into totipotent states leading eventually to plant regeneration (Tang *et al*., 2025).

**Fig. 4:**
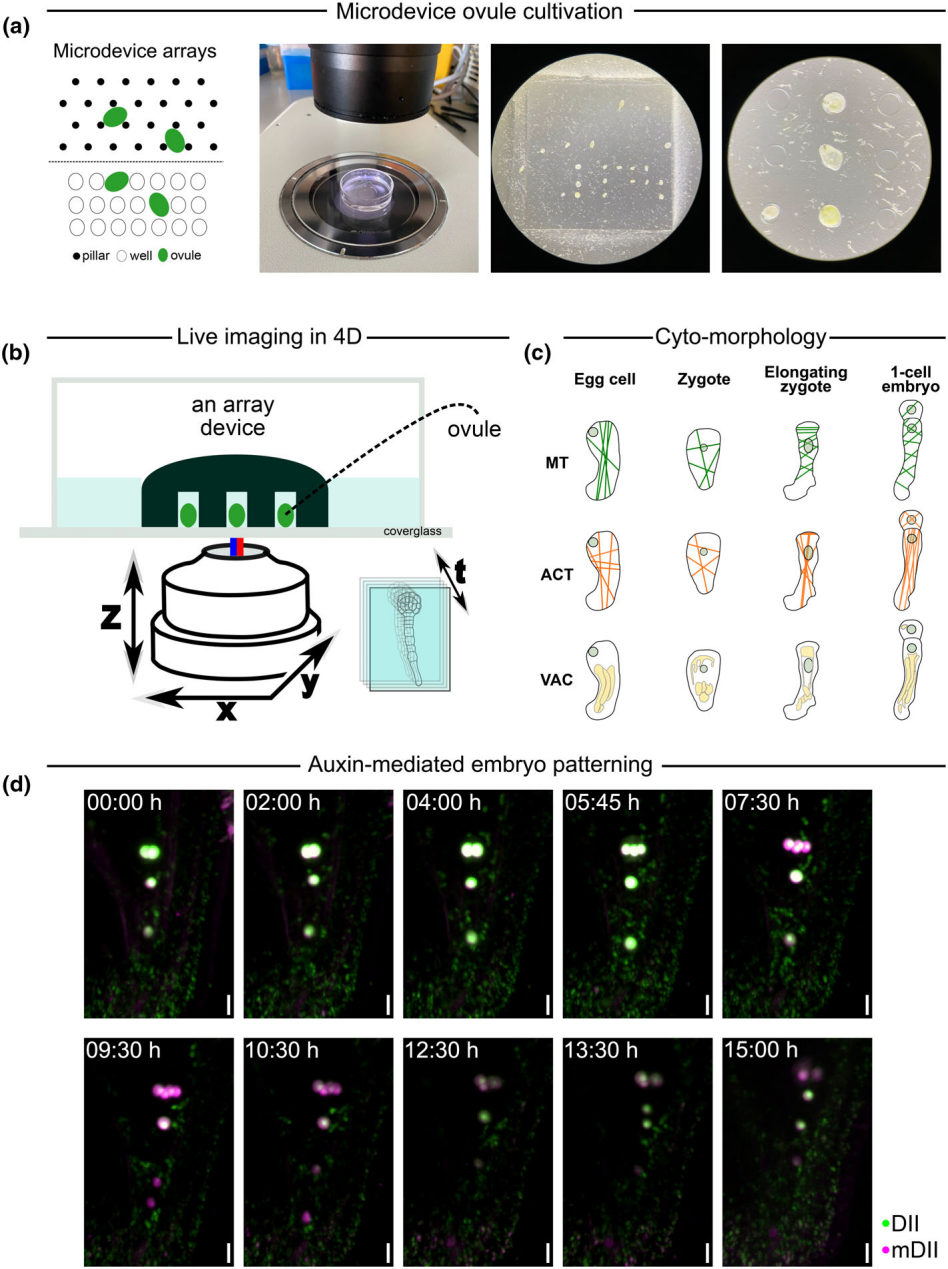
Live‐cell imaging of *Arabidopsis thaliana* embryos. (a) Design of ovule microdevices for live‐cell imaging of embryos. Left panel – examples of device array types (pillar‐based or well‐based); right panels show ovules positioned inside of well‐like devices under a stereomicroscope. (b) Schematic of a typical embryo live imaging setup on an inverted microscope. The array device is rotated to face the bottom of the glass‐bottom round dish. (c) Dynamics of subcellular components during the process of fertilisation from egg cell to the division of the zygote. Adapted from Radoeva *et al*. (2019). ACT, actin filaments; MT, microtubules; VAC, vacuoles. (d) Example of live imaging of the early embryo, taken on an inverted spinning disk microscope. The time‐lapse images show the auxin dynamics in the formation of the upper and lower proembryonic cell tiers between the 2‐ and 8‐cell stages. The auxin marker is a DII/mDII‐based ratiometric sensor driven by the *Ler* WOX2 promoter. Bars, 10 μm.

An important breakthrough was a method for long‐term imaging of *Arabidopsis* embryos growing inside ovules (Gooh *et al*., 2015). This experimental setup is based on a variation on the microfluidic polydimethylsiloxane (PDMS) device (Park *et al*., 2014) submerged in a trehalose‐containing Nitsch nutritional medium and observations made by a fluorescent microscope (typically multiphoton or spinning disk system) (Fig. 4a). This allows tracking embryos from the first zygotic division up until the heart stage, using long working‐distance objectives (Fig. 4b). Several important discoveries were made using this approach, for example, mapping the trajectories of apical and basal cell identities during divisions or confirmation that endosperm development is not necessary for initiation of cell fate specification in early embryos (Gooh *et al*., 2015). This setup has also been used to study embryo development in relation to processes such as suspensor‐derived embryogenesis (Radoeva *et al*., 2020), mitochondrial dynamics (Kimata *et al*., 2020), cytoskeleton dynamics (Kimata *et al*., 2016, 2023), and vacuolar distribution (Kimata *et al*., 2019) (Fig. 4c). In the same report (Gooh *et al*., 2015), this live imaging setup was coupled with a laser ablation system which is an impressive addition to the methodical toolbox of plant developmental biology. This system was used to precisely disrupt a single cell, for example, showing that after a disruption of an apical cell, the basal cell‐derived daughter cell directly underneath would change its identity becoming a new apical cell. This observation suggests the existence of embryonic apical dominance, whereby the apical cell inhibits the apical specification and proliferation of the basal cell. The possibility of long‐term imaging of embryos, coupled with an instrument such as single‐cell laser ablation, is exciting as it opens avenues to revisit old research questions and address new ones.

## Outlook and perspectives

Over the course of the past four decades, we have witnessed steady technological advances applied to plant embryogenesis; most obvious in imaging, genetics, and omics approaches. They largely capitalised on advances in the biomedical research and have harnessed the impressive progress of technologies there. Originally a specific niche for a few enthusiasts, the methodological developments in conjunction with landmark genetic screens focused on seedling and embryo patterning processes, followed by the molecular characterisation of corresponding genes in the 1990s, transformed *Arabidopsis* embryogenesis into a highly relevant model system generating key insights in fields of plant development, cell biology, and signalling. Since then, we are witnessing increasing interest in studying plant embryogenesis, transcending the classical *Arabidopsis* model systems and expanding to monocots as well.

Despite happening in an inherently complex setting of the developing seed, processes such as cell division orientation, cell polarisation and cell specification, when studied in embryos, typically occur in a setting of fewer cells and tissue types than during later developmental stages, thus providing an excellent model to address the mechanisms underlying these fundamental developmental processes. We can expect that the development of dynamic 3D geometry models and the use of cell fate‐specific markers will continue to be in focus of developmental studies in plant embryologists, as they have the clear potential to answer many questions related to cell division control and tissue patterning and their contribution to the formation of the body plan. On the other hand, applying live‐cell imaging methods to plant embryos is quite an intriguing possibility in its own right, and its highly technical nature likely helps advance this microscopy component of plant development research overall. In conjunction with genetics, hormone physiology and biophysics, live‐cell imaging has the potential to address long‐standing questions in many areas of plant developmental biology, beyond embryogenesis. The possibility of performing single‐cell laser ablations on living embryos would furthermore help to address other fundamental questions – mainly those related to cell‐to‐cell communication contributing to the establishment and maintenance of cell and tissue fates. The combination of these methods will also help to disentangle the multi‐layered communication pathways between the maternal integuments and the zygotic cells through the suspensor. Though this approach is technically demanding and has its limitations, as with everything concerning plant embryogenesis, persistence and dedication will lead to mastering and standardising methods such as these. Despite all technical challenges, ongoing methodological advances continue to expand experimental access to plant embryogenesis. In particular with genomes of virtually any potential land plant models within reach, the next frontiers are comparative and evolutionary embryogenesis. Thus, future progress in the plant embryology field will benefit from the integration of the emerging imaging, genetic, genomics, transcriptomic, and computational approaches, enabling more comprehensive analyses of plant embryo development.

## Competing interests

None declared.

## Author contributions

DB and JF conceived and planned the manuscript. DB, JF and MŽ contributed to writing, editing, and figure preparation. All authors have read and approved the final version of the manuscript.

## Disclaimer

The New Phytologist Foundation remains neutral with regard to jurisdictional claims in maps and in any institutional affiliations.
